# 3D virtual planning in orthognathic surgery and CAD/CAM surgical splints generation in one patient with craniofacial microsomia: a case report

**DOI:** 10.1590/2177-6709.21.1.089-100.oar

**Published:** 2016

**Authors:** Francisco Vale, Jessica Scherzberg, João Cavaleiro, David Sanz, Francisco Caramelo, Luísa Maló, João Pedro Marcelino

**Affiliations:** 1 Coordinator of Postgraduate Studies in Orthodontics, Universidade de Coimbra, Faculty of Dentistry, Department of Orthodontics, Coimbra, Portugal; 2 Postgraduate student, Universidade de Coimbra, Faculty of Dentistry, Department of Orthodontics, Coimbra, Portugal; 3 Maxillofacial Surgeon, Coimbra University Hospital Centre, Department of Maxillofacial Surgery, Coimbra, Portugal; 4 Auxiliar Professor, Universidade de Coimbra, Faculty of Dentistry, Institute for Biomedical Imaging and Life Sciences, Coimbra, Portugal; 5 Professor of Orthodontics, Universidade de Coimbra, Faculty of Dentistry, Department of Orthodontics, Coimbra, Portugal

**Keywords:** Orthognathic surgery, CAD/CAM, 3D surgical splint

## Abstract

**Objective:**

In this case report, the feasibility and precision of tridimensional (3D) virtual planning in one patient with craniofacial microsomia is tested using Nemoceph 3D-OS software (Software Nemotec SL, Madrid, Spain) to predict postoperative outcomes on hard tissue and produce CAD/CAM (Computer Aided Design/Computer Aided Manufacturing) surgical splints.

**Methods:**

The clinical protocol consists of 3D data acquisition of the craniofacial complex by cone-beam computed tomography (CBCT) and surface scanning of the plaster dental casts. The ''virtual patient'' created underwent virtual surgery and a simulation of postoperative results on hard tissues. Surgical splints were manufactured using CAD/CAM technology in order to transfer the virtual surgical plan to the operating room. Intraoperatively, both CAD/CAM and conventional surgical splints are comparable. A second set of 3D images was obtained after surgery to acquire linear measurements and compare them with measurements obtained when predicting postoperative results virtually.

**Results:**

It was found a high similarity between both types of surgical splints with equal fitting on the dental arches. The linear measurements presented some discrepancies between the actual surgical outcomes and the predicted results from the 3D virtual simulation, but caution must be taken in the analysis of these results due to several variables.

**Conclusions:**

The reported case confirms the clinical feasibility of the described computer-assisted orthognathic surgical protocol. Further progress in the development of technologies for 3D image acquisition and improvements on software programs to simulate postoperative changes on soft tissue are required.

## Abstract (pt)

**Objetivo:**

neste relato de caso, de um paciente com microssomia craniofacial, testou-se a viabilidade e a precisão do planejamento virtual tridimensional (3D) utilizando o *software* Nemoceph 3D-OS (Software Nemotec SL, Madri, Espanha) para prever os resultados pós-operatórios em tecidos duros e produzir *splints* cirúrgicos CAD/CAM.

**Métodos:**

o protocolo clínico usado consistiu na aquisição de dados 3D do complexo craniofacial por meio de tomografia computadorizada de feixe cônico (TCFC) e digitalização dos modelos de gesso. O "paciente" criado virtualmente foi submetido à cirurgia virtual e obteve-se uma simulação dos resultados pós-operatórios nos tecidos duros. Os *splints*cirúrgicos foram confeccionados usando-se a tecnologia CAD/CAM, e permitiram que o planejamento cirúrgico virtual fosse transferido para a sala cirúrgica. No transoperatório, tanto os *splints* CAD/CAM quanto os *splints* cirúrgicos convencionais mostraram-se similares. Um segundo conjunto de imagens 3D foi obtido após a cirurgia, para que medidas lineares pudessem ser obtidas e comparadas com as medidas feitas na simulação virtual dos resultados pós-operatórios.

**Resultados:**

foi encontrada uma forte semelhança entre os dois tipos de*splints* cirúrgicos, que se adaptaram da mesma forma nas arcadas dentárias. As medidas lineares mostraram algumas discrepâncias entre os resultados cirúrgicos reais e os resultados previstos na simulação virtual 3D; porém, a análise desses resultados requer cautela, em virtude da presença de inúmeras variáveis que podem neles interferir.

**Conclusões:**

o caso relatado no presente estudo confirma a viabilidade clínica do protocolo descrito de cirúrgica ortognática assistida por computador. Porém, ainda se faz necessária uma maior evolução no desenvolvimento de tecnologias para a aquisição de imagens 3D e nos *softwares* que simulam as alterações pós-operatórias nos tecidos moles.

## INTRODUCTION

Comprehensive visualization and records of the craniofacial complex are important goals in orthodontic imaging which have been conventionally achieved by means of plaster dental casts, photographs and radiographs. However, cone-beam computed tomography (CBCT) has gained considerable acclaim worldwide as a viable tridimensional (3D) imaging modality.^1^ CBCT is a medical image acquisition technique based on a cone-shaped X-ray beam centered on a two-dimensional (2D) detector. The scanning software collects the raw image data and reconstructs them into a 3D data set.^2^ Whenever it is necessary to comprise the whole craniofacial region in the study, as in cases of cephalometry analyses, a large field of view (FOV) must be selected, which, according to the American Academy of Oral and Maxillofacial Radiology,^3^ captures a spherical volume diameter or cylinder height greater than 15 cm.

The first computer-based cephalometric systems appeared in the late 1970s, and since then several two-dimensional (2D) computer-assisted imaging systems were created, allowing for a combination of photographs, tracings, and radiographs. These computer-assisted programs permit rapid measurements and treatment planning, but the validity and reliability of these systems are limited by their 2D nature when dealing with a 3D structure.^4^

In a reconstruction of a CBCT scan, the skin is untextured and the dental structures may contain streak artifacts caused by restorations or orthodontic fixed appliances. Therefore, it is necessary to superimpose a textured facial soft tissue surface (mapping 2D photographs, a 3D photograph or a 3D surface scan with the reconstruction of CBCT data) and upgrade the dental images (by digitization of the plaster cast, or scan of the dental impression with a CBCT or a surface laser scanner; or an impression obtained by direct intraoral 3D scanning devices), thereby improving the visualization of the interocclusal relationship with precise dental morphology of the surfaces and cusps.^5^

In orthognathic surgery, the repositioning of skeletal constructs is conventionally guided by the surgical splint technique obtained through time-consuming and imprecise model surgery.^6^^,^ 7 The 3D fusion model mentioned above replaces the need for model surgery, since the virtual head, besides the diagnosis and treatment plan, can also be used to design a surgical splint.^5^

In the traditional method, in order to achieve a precise diagnosis of the dentoskeletal deformity and create a treatment plan intraoperatively reproducible, it is necessary to collect data from different sources, such as photographs, cephalograms, dental casts, physical examination along with face bow record and its transfer to semi-adjustable articulators, and measurement of plaster casts movement, according to the surgical simulation.^6^^,^ 7 ^,^ 8 Therefore, despite being an established and accepted method, a detailed analysis of the traditional model surgery technique reveals that theoretically it suffers from several sources of error and inaccuracy, with insufficient control of movements, such as rotation and translation, with regard to the whole cranial situation.^9^

As a result of developments in 3D imaging technology, surgeons are provided with extra information that could not be obtained from lateral cephalogram alone, thus improving the quality of preoperative planning.^8^^,^ 10 Multiple software programs are available for 3D planning, allowing an interaction with 3D images to simulate surgery and visualize the prediction of postoperative outcomes in soft and hard tissues. Surgical splints, manufactured using computer-aided design/computer-aided manufacturing (CAD/CAM) technology, have been developed to avoid errors in the traditional model process that can lead to suboptimal outcomes.^6^^,^ 8

In this case report, the feasibility and precision of 3D virtual planning in one patient with craniofacial microsomia is tested using Nemoceph 3D-OS software (Software Nemotec SL, Madrid, Spain) to predict postoperative outcomes on hard tissue and produce CAD/CAM surgical splints.

## CASE REPORT

**Figure 1:**
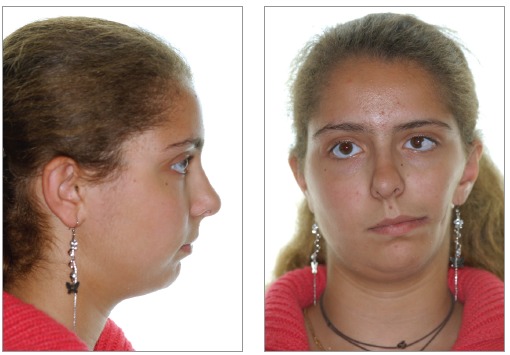
- Lateral and frontal extraoral photographs before orthodontic treatment. Patient at 14 years of age.

**Figure 2:**
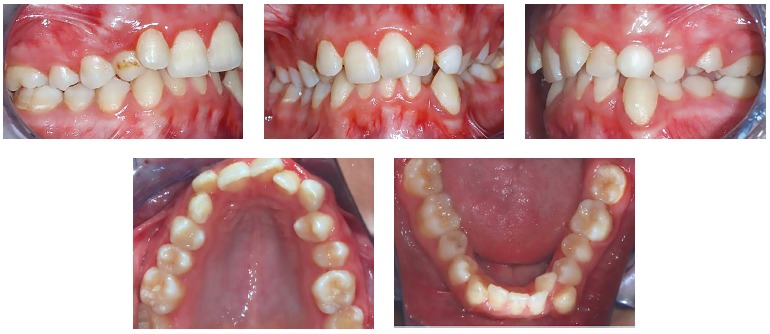
- Intraoral photographs before orthodontic treatment. Patient at 14 years of age.

A 19-year-old female presented with left craniofacial microsomia Pruzansky IIA associated with diminished left jugal soft tissues, a unilateral cleft lip and macrostomia, maxillary and mandibular retrusion and vertical maxillary excess, requiring combined orthodontic-orthognathic surgery treatment. Extraoral and intraoral initial status, previous to orthodontic treatment, can be seen in Figures 1 and 2, respectively.

**Figure 3:**
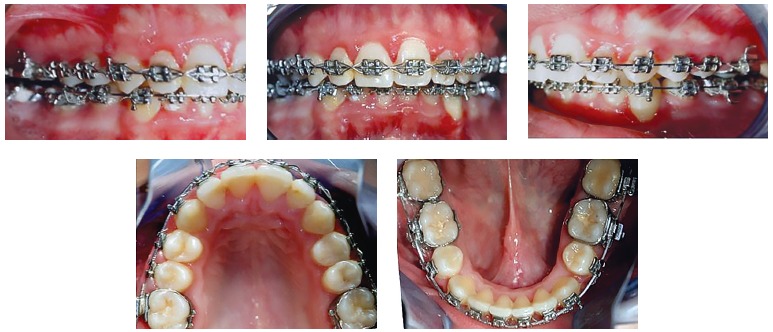
- Intraoral photographs after preoperative orthodontic treatment.

Preoperative orthodontic treatment was performed with a multibracket appliance (Roth prescription, slot size 0.018 x 0.030-in, with Ni-Ti archwires followed by SS archwires) and extraction of mandibular first premolars for correction of dental compensations, enabling the occlusal coordination of both dental arches (Fig 3). Surgical treatment objectives were maxillary advancement of 3 mm, anterior impaction of 6 mm, impaction of 8 mm at the level of the right canine and 4 mm at the level of the left canine, with Le Fort I osteotomy and anterior and counterclockwise mandibular repositioning with bilateral sagittal split osteotomy (BSSO). The surgical plan and acrylic surgical splints generated by conventional planning methods were available as backup during surgery.

**Figure 4:**
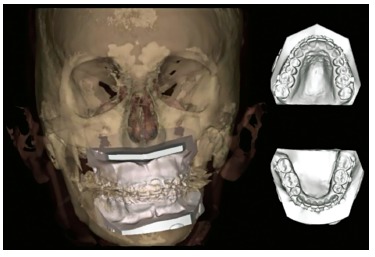
- 3D image of the skull fused with surface scans of the upper and lower plaster dental casts.

Two CBCT scans were obtained: one preoperative (two months prior to orthognathic surgery) and one postoperative (one month after surgery), using the i-CAT^TM^ device, version 17-19 (Imaging Sciences International, Hatfield, Pa, USA) with a FOV set to a height of 17 cm and a diameter of 23 cm. The radiological parameters used were 120 kV of tube voltage, 5 mA of tube current and exposure of 37.10 mAs, with a voxel size of 0.3 x 0.3 x 0.3 mm. The images obtained using CBCT were stored in DICOM format and sent to Nemotec CAD/CAM Centre (Centro Integrado Investigación, Madrid, Spain) together with patient's dental plaster casts and digital facial photographs (frontal, oblique and lateral). Upper and lower dental plaster casts were scanned using a surface laser scanner and incorporated in the 3D image by means of a semi-automatic procedure (Fig 4). Segmentation and conversion from DICOM to 3D images were carried out using Nemoceph 3D-OS software.

At first, the whole skull data set was manually aligned to natural head position (NHP), using patient's photographs (captured in NHP - a standardized and reproducible position of the head in an upright relaxed posture, with the eyes focused on a point in the distance at eye level^11^) to help in the orientation of the 3D skull data set. 

**Figure 5:**
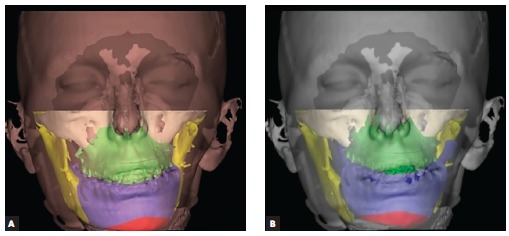
- 3D image of the preoperative situation (A) and the postoperative virtual simulation of the predicted results on hard tissues after repositioning the mobilized bone structures (B).

With the aid of the 3D images, it was possible to perform virtual osteotomies using predefined Le Fort I and BSSO lines. The repositioning of the osteotomized bone structures was performed for the maxilla according to the conventional treatment plan defined by clinical examination, whereas for the mandible it was performed through a semi-automatic procedure and some manual adjustments in order to simulate correct final teeth intercuspation. A 3D simulation of postoperative results on hard tissue was produced (Figs 5A, 5B). The mirror imaging technique, which reflects the nonaffected side of the skull on a given symmetry plane, is a useful method to visualize, evaluate and measure asymmetries (as in cases of craniofacial microsomia). It can also serve as a template for reconstructions, but since no surgical reconstruction plates or grafts were used, the reposition of the mandible could be effectively obtained with the final correct teeth intercuspation position. 

**Figure 6:**
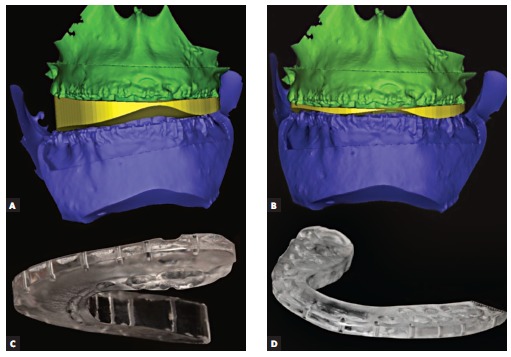
- 3D planned intermediate (A) and final (B) situation with the virtual splint set between the dental arches. Intermediate (C) and final (D) CAD/CAM surgical splints milled on PMMA.

Treatment plan was transferred to the patient by means of intermediate and final CAD/CAM surgical splints (for repositioning of the maxilla and mandible, respectively) which were generated in the computer and fabricated by a milling machine milling the surgical splints on polymethyl methacrylate (PMMA) (Figs 6A , 6B).

**Figure 7:**
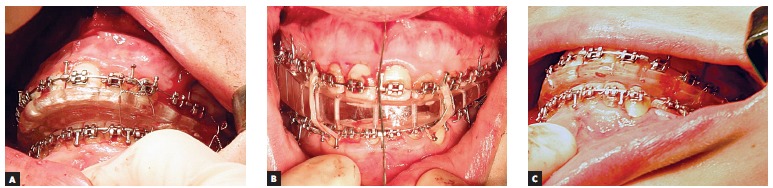
- Intraoperative situation of the conventional initial splint (A), intermediate CAD/CAM splint (B) and final CAD/CAM splint (C) fitting the dental arches.

**Figure 8:**
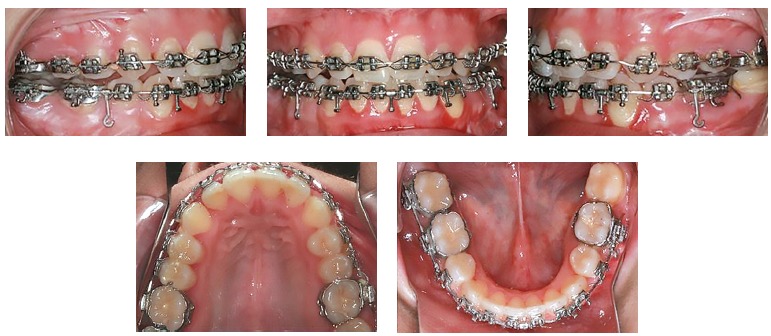
- Intraoral photographs of the unfinished postoperative orthodontic treatment, 10 months after orthognathic surgery.

**Figure 9:**
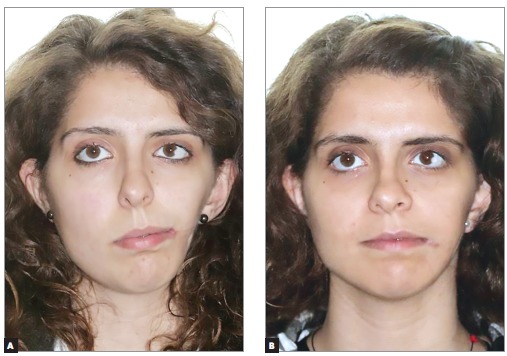
- Frontal facial photographs at rest, before (A) and after orthognathic surgery, comissuroplasty and jugal lipofilling (B).

**Figure 10:**
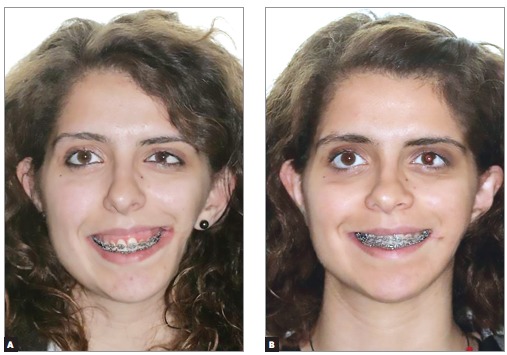
- Frontal facial photographs at smiling, before (A) and after orthognathic surgery, comissuroplasty and jugal lipofilling (B).

Bimaxillary surgery was performed under general anesthesia, with Le Fort I and BSSO osteotomies. Intraoperative registration of the correct condylar position within the glenoid fossa was performed using the initial splint (acrylic splint generated by the conventional method), and the reposition of the osteotomized maxilla and mandible was guided by the CAD/CAM surgical splints using fixation plates and screws (Figs 7A-C). Maxillomandibular elastic fixation was maintained for six weeks after surgery, allowing proper bone consolidation. Treatment continued with the postoperative orthodontic phase for final occlusal detailing (Fig 8). Six months after orthognathic surgery, left comissuroplasty and jugal lipofilling with Coleman's technique were performed using an autologous abdominal fat graft (Figs 9A, 9B and 10A, 10B).

## RESULTS

The CAD/CAM splints were clinically evaluated by an experienced clinician regarding its adaptation, passivity and occlusal scheme. There was no need for adjustments, as a perfect fit was obtained during splints try-in performed in the patient's mouth before surgery.

Conventional intermediate and final acrylic splints try-ins were also performed intraoperatively, after reposition and fixation of the jaws with CAD/CAM splints, and an equal fitting was verified with both types of splints, demonstrating that both splints were able to transfer the same surgical plan to the patient at the time of surgery.

**Figure 11:**
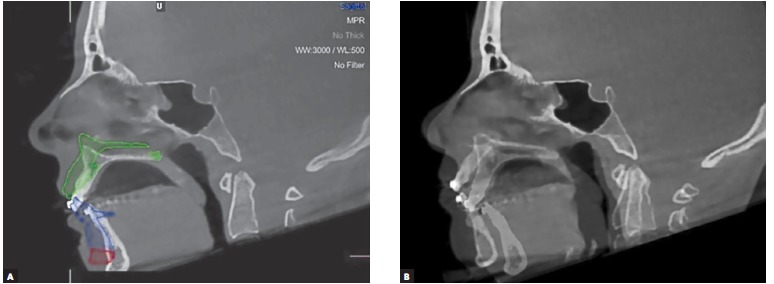
- Sagittal view of the preoperative situation and virtual postoperative simulation (green, blue and red tracing), performed on Nemoceph 3D-OS software (A); pre- and postoperative CBCT was registered based on the cranial base surface with 3D Slicer 4,1 software (B).

**Figure 12:**
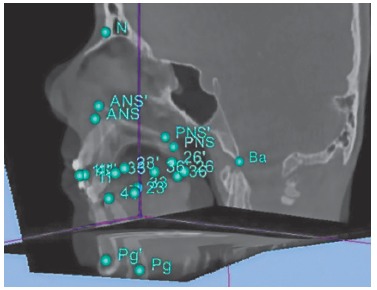
- Display of the three planes (sagittal, axial and coronal) of the registered pre- and postoperative CBCTs, with 3D distribution of landmarks on 3D Slicer 3,6 software.

Measurements were performed in the 3D simulation using Nemoceph 3D-OS software for assessment of quantitative changes between the preoperative and the postoperative simulated positions of selected dental and bone landmarks (Fig 11A). A fully automated voxel-based registration was computed by the open-source software 3D Slicer 4.1 (The Slicer Community) that optimally aligned pre- and postoperative CBCT's at the cranial base surface, as these structures are not altered by surgery (Fig 11B). The same measurements were performed in this image set to assess the difference between preoperative and actual postoperative positions of the same dental and bone landmarks, using the open-source software 3D Slicer 3.6 (The Slicer Community). Linear measurements obtained (Table 1) demonstrate the displacement of some selected landmarks from the preoperative situation to the postoperative outcome (predicted in the 3D surgical simulation and actually obtained after surgery). These measurements were performed in the X-axis (depth), Y-axis (width, deviation) and Z-axis (height), for dental landmarks (incisal edge of #11, 41; cusp tip of #13, 23, 33, 43; mesiobuccal cusp of 16, 26, 36, 46) and bone landmarks (Pogonion, Anterior Nasal Spine and Posterior Nasal Spine) (Fig 12). At the time of the second CBCT acquisition, there was still some postoperative soft tissue swelling, and since the software used does not provide soft tissue simulation, the evaluation of the outcomes was only performed for hard tissues.

**Table 1:** - Displacement of selected landmarks from the preoperative situation to the postoperative outcome (predicted in the 3D surgical simulation and actually obtained after surgery),

**Linear measurements**		**Difference between preoperative and predicted postoperative positions (mm)(Nemoceph 3D-OS)**	**Difference between preoperative and actual postoperative positions (mm)(3D Slicer 3.6)**	**Discrepancy between the predicted and actual outcome (mm)**
Height 11		+6.5	+10.3	3.8
Height 13		+6.7	+11.5	4.8
Height 23		+4.1	+6.4	2.3
Height 16		+9.2	+9.5	0.3
Height 26		+3.4	+3.3	0.1
Height 41		+3.6	+5.8	2.2
Height 33		+1.3	+2.6	1.3
Height 43		+5.3	+5.8	0.5
Height 36		+0.6	+0.1	0.5
Height 46		+7	+7.6	0.6
Mx. Midline Dev.		0	-0.2	0.2
Width 13		-0.2	-0.5	0.3
Width 23		+0.4	+2.8	2.4
Width 16		-0.7	-0.1	0.6
Width 26		+1.1	+0.7	0.4
Md. Midline Dev.		-0.3	-0.4	0.1
Width 33		+0.5	+0.6	0.1
Width 43		-0.1	-0.7	0.6
Width 36		+2.2	+1.1	1.1
Width 46		-1.3	-0.2	1.1
Depth 11		+3	+8.4	5.4
Depth 13		+2.4	+10.8	8.4
Depth 23		+3	+7.5	4.5
Depth 16		+3	+9.1	6.1
Depth 26		+3	+6.6	3.6
Depth 41		+8.7	+14.7	6
Depth 33		+10	+13.2	3.2
Depth 43		+7.9	+14.3	6.4
Depth 36		+10.7	+14.2	3.5
Depth 46		+7.2	+10.9	3.7
Pg	Height	+ 2,7	+3,4	0.7
	Dev.	-5	-3	2
	Depth	+8,6	+16	7.4
ANS	Height	+4,8	+ 5,3	0.5
	Depth	+ 1,7	+ 0,2	1.5
PNS	Height	+ 5,6	+ 3,8	1.8
	Depth	+ 6,2	+ 5,2	1

Pogonion (Pg): most anterior midpoint of the chin on the outline of the mandibular symphysis. Anterior Nasal Spine (ANS): most anterior midpoint of the anterior nasal spine of the maxilla. Posterior Nasal Spine (PNS): most posterior midpoint of the posterior nasal spine of the palatine bone. Maxillary midline deviation (Mx Midline Dev) Mandibular midline deviation (Md Midline Dev). Height (vertical movement): (+) upward postsurgical position. Width (transversal movement): (+) distalization of the postsurgical position in relation to the facial midline, (-) mesialization of the postsurgical position in relation to the facial midline. Depth (anteroposterior movement): (+) advancement of the postsurgical position.

## DISCUSSION

Modern 3D virtual planning for orthognathic surgery has critical advantages compared to conventional treatment planning. Simulating the operation on plaster dental casts is difficult, especially in cases of complicated two-jaw surgery, as it requires many laboratory-based steps that are time-consuming and may lead to potential errors.^12^ Furthermore, the splint which transfers the final relative position of the maxilla to the mandible, summates all of the errors of the previous stages.^13^ This emerging technology allows us to replicate more closely the actual patient while providing access to more and higher-quality information about patient's 3D anatomy and improving the ability to identify conditions that are not detectable with 2D conventional imaging techniques, thus improving the accuracy and reliability of diagnosis and treatment.^14^ Moreover, unlike conventional model surgery on dental casts, this technology allows to virtually perform multiple simulations of different osteotomies and skeletal movements in order to evaluate multiple surgical plans.^5^^,^ 15

Surgical splints can be manufactured with rapid prototyping techniques in order to transfer the virtual plan to the operating room. The accuracy of rapid prototyping procedures for orthognathic surgery is now beyond all question, and the reliability of these CAD/CAM-generated splints has already been validated.^8^^,^ 9 ^,^ 15 ^-^ 20 The approaches described by other authors^9^^,^ 15 ^,^ 16 ^,^ 17 ^,^ 18 ^,^ 20 differ from the one used in this clinical case in the way of obtaining data as well as in the type of software and hardware used, which makes it difficult to compare the different 3D virtual planning systems. The high similarity found between CAD/CAM and conventional surgical splints in this case allows us to confirm that the CAD/CAM method is a valid and reliable technique for designing surgical splints that will accurately reproduce our 3D virtual planning in the operating room.

 Treatment outcome evaluation is possible through techniques of voxel-based rigid registration and superimposition on a 3D reference system.^15^ These recent software tools allow an optimal alignment of 3D CBCT data sets, avoiding observer-dependent traditional techniques based on overlap of anatomic landmarks.^21^

Using this methodology, treatment plans can be stored in the computer where patient's records are instantly available, and the dental casts can be discarded after digitalization, saving the space normally taken up by physical elements used in conventional planning.^8^^,^ 9 Diagnosis can be shared with the patient using a 3D image that can be easily understood, and helps providing clear and realistic pretreatment informed consent.^8^Moreover, this methodology gathers the greatest advantages offered by telemedicine, as all preoperative information can be easily shared with colleagues in any part of the world, thereby facilitating communication and shared decision making.^5^^,^ 8 ^,^ 22

Regarding the limitations of this technique, the most problematic inconvenience is probably the fact that although there are advanced 3D imaging techniques capable of individually displaying the facial skeleton, dentition, and soft tissues, there is currently no single imaging technique that can accurately capture the complete triad with optimal quality for orthognathic surgery planning.^15^ The software used in the clinical case reported herein does not allow virtual soft tissue simulation at all; however, there are commercially available programs which use spring deformation and morphing programs for soft tissue surgical predictions.^23^ This is not biomechanically accurate, nor has it been validated.^24^^,^ 25 ^,^ 26 The usage of an inhomogeneous biomechanical model, which distinguishes the mechanical behavior of fatty and muscle tissue and considers individual soft tissue thicknesses and properties in the model generation, should be further investigated to optimize the validity and reliability of the procedure of soft tissue simulation.^25^^,^ 26 These improvements would be of great value considering the large number of patients undergoing this type of surgery for aesthetic reasons.^4^^,^ 8 Since, at the moment, soft tissue prediction is still unclear, the clinician should be careful in communicating this information to the patient.^18^ Furthermore, the clinician must not forget that 3D virtual models are, despite their accuracy, a static representation of patient's tissues at the point of image capture. Hence, detailed physical examination is still absolutely essential in order to obtain extremely valuable dynamic information for precise orthognathic surgery planning.^15^ In this clinical case, during virtual repositioning of the maxilla, movements of impaction and advancement were performed according to the conventional treatment plan obtained by clinical examination. That was necessary, since the software does not enable soft tissue simulations and, consequently, does not allow guidance of jaw movements through visualization of soft tissue corresponding changes until the intended profile projection and the ideal exposure of maxillary anterior teeth are achieved. 

Considering the postsurgical prediction results in this clinical case, the linear measurements shown in Table 1 reflect some discrepancies between the actual surgical outcomes and the predicted results from the 3D virtual simulation. Caution must be taken in the analysis of these results. There are several variables which can affect the results, such as difficulty locating and placing some 3D anatomic landmarks using CBCT-generated volumetric images and slices, variables inherent to surgical procedures and the influence of postsurgical relapse on hard tissues. The discrepancies found particularly in the anterior region of the maxillomandibular complex (reflected in the measurements for anterior teeth (#11, 13, 23, 41, 33, 43) might be explained with an excessive anterior maxillary bone removal after Le Fort I osteotomy, leading to a non-predicted excessive mandibular counterclockwise rotation, perceived in excessive Pogonion (Pg) advancement. This might be explained with the impossibility of controlling vertical movement of the maxilla with the surgical splint technique, which transfers the entire 3D virtual repositioning of the maxilla (including rotations, translations, and leveling), except for its vertical position to the cranial base.^18^ In recent literature, a new approach has been described using individually designed templates that are placed on the fixed maxilla instead of the movable mandible,^6^^,^ 13 ^,^ 27 ^,^ 28 which eliminates potential errors caused by autorotation of the mandible, relocating the maxilla independently. 

In order to enable this paradigm shift in routine planning of orthognathic surgery, both image acquisition systems and 3D virtual planning software must become user-friendly, easily accessible and available at a relatively low cost.^18^

## CONCLUSIONS

The reported case confirms the clinical feasibility of a computer-assisted orthognathic surgical protocol incorporating virtual planning and its transfer to the operating room using CAD/CAM fabricated surgical splints. 

Further progress is required in the development of technologies for 3D image acquisition and improvements on software programs to simulate postoperative changes on soft tissues.
